# Inhibitors of the Wnt pathway in osteoporosis: A review of mechanisms of action and potential as therapeutic targets

**DOI:** 10.17305/bb.2024.11200

**Published:** 2024-11-25

**Authors:** Jiayi Song, Weirong Chang, Yujie Wang, Peng Gao, Jie Zhang, Zhipan Xiao, Fangyu An, Chunlu Yan

**Affiliations:** 1 School of Basic Medicine, Gansu University of Chinese Medicine, Lanzhou, China; 2 School of Tradional Chinese and Werstern Medicine, Gansu University of Chinese Medicine, Lanzhou, China; 3 Teaching Experiment Training Center, Gansu University of Chinese Medicine, Lanzhou, China

**Keywords:** Wnt signaling pathway, osteoporosis, sclerostin, DKK1, WIF1, SFRP, bone metabolism

## Abstract

The Wnt signaling pathway is one of the most important and critical signaling pathways for maintaining cellular functions, such as cell proliferation and differentiation. Increasing evidence substantiates that the Wnt signaling pathway also plays a significant role in the regulation of bone formation in osteoporosis. Accordingly, inhibitors of this pathway, such as sclerostin, Dickkopf-1 (DKK1), WNT inhibitory factor 1 (WIF1), and secreted frizzled-related proteins (SFRPs), have a negative regulatory role in bone formation and may serve as effective therapeutic targets for osteoporosis. This review examines the mechanisms of action of Wnt signaling pathway inhibitors in osteoporosis, the relationship between the Wnt pathway and its inhibitors, and new molecular targets for osteoporosis treatment. Overall, the regulatory mechanisms of Wnt pathway inhibitors are summarized to provide scientific and theoretical guidance for the treatment and prevention of osteoporosis.

## Introduction

Osteoporosis is the most common chronic metabolic bone disease, characterized by low bone mass, decreased bone density, and deterioration of bone microstructure [1]. These detriments increase the risk of bone fragility and fractures [2]. Under normal physiological conditions, bones maintain a dynamic and balanced state, with the continuous generation of new bone and resorption by osteoclasts. With aging, this dynamic balance is disrupted, and bone resorption gradually exceeds formation, leading to osteoporosis [3]. Potential factors contributing to the development of osteoporosis include aging, reduced oestrogen levels, and chronic inflammation, which collectively increase the risk of fractures in affected patients [4–6]. Globally, more than 75 million people have osteoporosis [7]. As the population ages, medical expenditures for osteoporotic fractures are projected to increase by more than 50%, from $17 billion in 2005–2025, placing a heavy economic burden on society and families [8, 9].

Current treatments for osteoporosis include bone formation promoters, such as teriparatide and romosozumab, and bone resorption inhibitors, such as oestrogen and bisphosphonates. However, the long-term use of these drugs is associated with a series of side effects, including myocardial infarction, liver and kidney injury, and endometrial cancer [10–12]. Therefore, identifying new targeted therapies is pivotal for the clinical treatment of osteoporosis. The role of the Wnt signaling pathway in osteoporosis has garnered considerable attention due to in-depth studies on signaling pathways. The Wnt pathway is critical [13, 14] for regulating bone homeostasis by promoting osteoblast formation and inhibiting osteoclast resorption [15]. This pathway also induces the osteogenic differentiation of mesenchymal stem cells (MSCs) and their progression to mature osteoblasts [16]. The Wnt pathway has been confirmed to play a significant role in the regulation of osteoporosis.

Recent evidence [17] indicates that inhibitors of the Wnt signaling pathway can negatively regulate osteogenesis. These inhibitors include sclerostin, Dickkopf-1 (DKK1), WNT inhibitory factor 1 (WIF1), and secreted frizzled-related proteins (SFRPs). These inhibitors primarily bind to Wnt—the ligand of the Wnt signaling pathway—Frizzled (FZD), and low-density lipoprotein receptor-related protein 5/6 (LRP5/6), thereby inhibiting the expression of Wnt, β-catenin, and other key proteins in the pathway. This inhibition leads to the downregulation of T-cell-specific transcription factor 1 (TCF-1), Runt-related transcription factor 2 (Runx2), alkaline phosphatase (ALP), and osteopontin (OPN), which are downstream osteogenic markers, thereby promoting osteoporosis [18].

Additionally, other studies [19, 20] have found that these inhibitors can suppress the expression of key proteins in the Wnt signaling pathway, such as Wnt and β-catenin, by binding to the cell membrane receptors FZD and LRP5/6. This significantly increases the ratio of receptor activator of nuclear factor kappa beta ligand (RANKL) to osteoprotegerin (OPG), as well as the number of osteoclasts. Tartrate-resistant acid phosphatase (TRAP)-positive osteoclasts are significantly increased in rat models. Therefore, blocking the expression of Wnt signaling pathway inhibitors, such as sclerostin, DKK1, WIF1, and SFRPs, can effectively promote bone formation and inhibit bone resorption. These inhibitors are expected to represent promising targets for the treatment of osteoporosis.

In this review, the Wnt signaling pathway, including the role and regulatory mechanisms of its inhibitors in osteoporosis, is discussed. In particular, the effects of small molecules targeting non-coding RNA, natural medicines, monomer components of traditional Chinese medicine, and Western medicine on the expression of Wnt signaling pathway inhibitors were analyzed, and the potential mechanisms of their curative effects on osteoporosis were examined. Overall, this review seeks to highlight the regulatory effects of Wnt signaling pathway inhibitors on osteoblasts and osteoclasts in osteoporosis.

## Wnt signaling pathway and its role in osteoporosis

The term “Wnt” is a fusion of the Int1 proto-oncogene, located at the integration site of a mouse breast tumor virus, and the homologous wingless gene of Drosophila [21]. The Wnt signaling pathway is highly conserved and plays an important role in multicellular organisms [22], maintaining bone homeostasis by promoting bone formation and inhibiting bone resorption [14]. This pathway, which comprises both classical and non-classical pathways, induces osteogenic differentiation and maturation of mesenchymal stem cells (MSCs) [23] and regulates bone homeostasis in osteoporosis. The non-classical Wnt signaling pathway includes the Wnt/Planar cell polarity (PCP) and Wnt/Ca2+ pathways [24, 25]. The Wnt signaling pathway is closely related to the occurrence and development of osteoporosis [26–28].

### Classical Wnt/β-catenin signaling pathway

**Figure 1.**
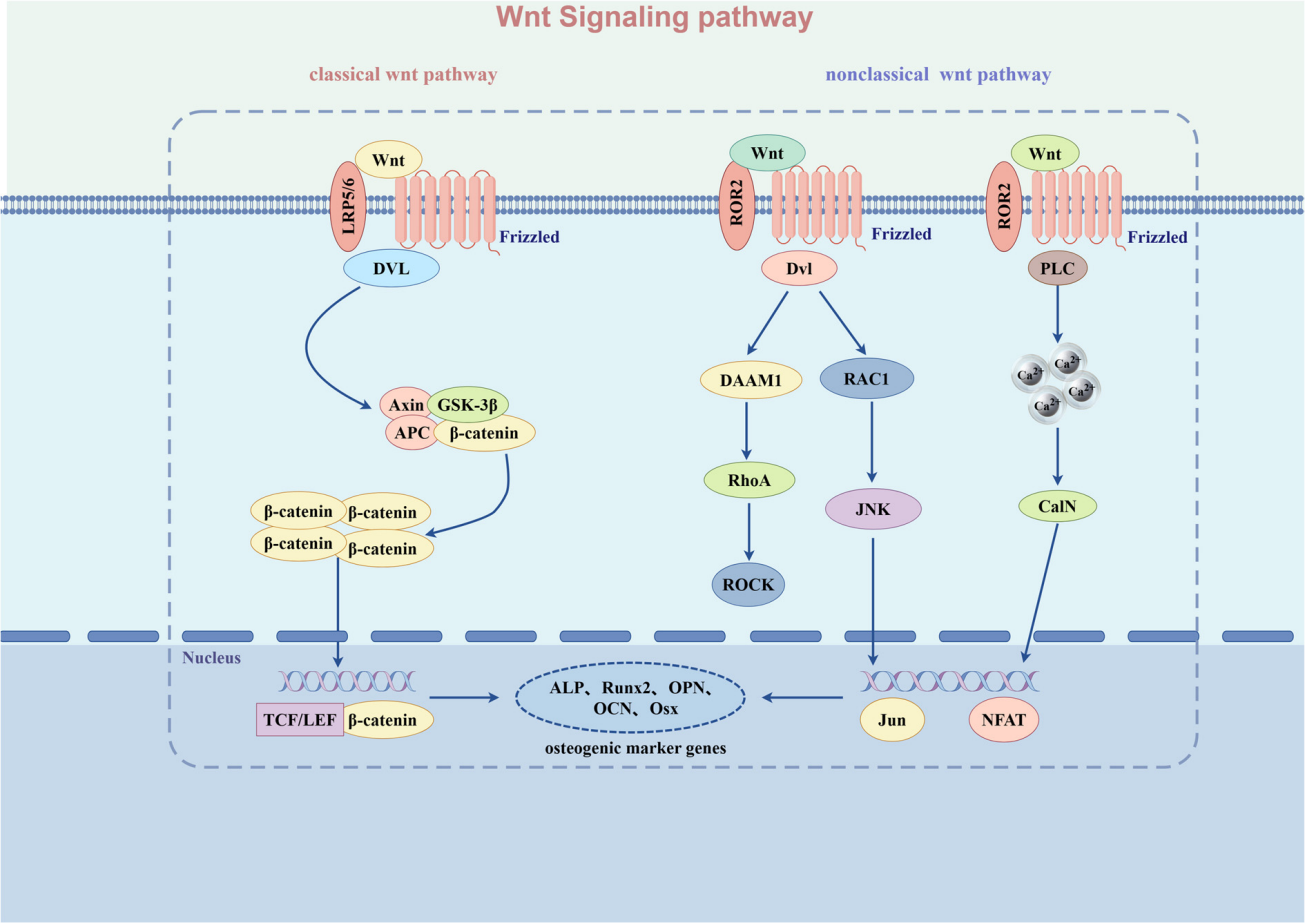
**Regulatory mechanisms of the Wnt signaling pathway.** Wnt signaling occurs through the activation of the Frizzled (FZD) receptor and the co-receptor LRP5/6 or ROR2 by the binding of Wnt proteins. The Wnt signaling pathway is divided into the classical Wnt/β-catenin signaling pathway and the non-classical Wnt/PCP and Wnt/Ca2+ signaling pathways. LRP5/6: Lipoprotein receptor-related protein 5/6.

The classical Wnt signaling pathway consists of the extracellular Wnt ligand, FZD, LRP5/6, cytoplasmic β-catenin, disheveled (DVL), adenomatous polyposis coli protein (APC), glycogen synthase kinase 3β (GSK-3β), axin inhibitor protein (Axin), and nuclear T cell factor/lymphoid enhancer-binding factor (TCF/LEF) transcription factor [29–31]. Wnt protein binds to FZD and LRP5/6 receptors on the cell membrane [32]. The cytoplasmic portion of FZD is activated upon interaction with DVL, which then inhibits the Axin-GSK-3β-APC complex, preventing β-catenin phosphorylation. As β-catenin accumulates in the cytoplasm, it translocates to the nucleus [33] and binds to TCF/LEF, activating the downstream Wnt signaling pathway [34, 35] (Figure 1).

The classical Wnt/β-catenin signaling pathway is closely related to the occurrence and development of osteoporosis. Wang et al. [36] determined the expression levels of differentiation antagonizing non-protein coding RNA (DANCR), microRNA (miR)-320a, and catenin beta 1 (CTNNB1, which codes for β-catenin), in patients with osteoporosis [36]. The expression levels of DANCR and miR-320a were found to be relatively high, while that of CTNNB1 was low. Moreover, during osteoblastic differentiation induced by bone marrow MSCs (BMSCs), the expression levels of DANCR and miR-320a were significantly decreased, whereas CTNNB1 expression increased [36]. The mRNA and protein levels of osteogenic markers, Runx2, OPN, osteocalcin (OCN), and β-catenin, in ALP and BMSCs of the miR-320a inhibitor group were also significantly higher than those in the control group; the overexpression of miR-320a resulted in the opposite outcome [36]. These findings indicate that the expression levels of DANCR and miR-320a are higher in patients with osteoporosis, and the inhibition of miR-320a expression can significantly upregulate the expression of bone-related markers, including Runx2, OPN, and OCN, alongside key target molecules, β-catenin and TCF-1, in the Wnt/β-catenin signaling pathway, thereby promoting bone formation.

Additionally, the expression of forkhead box protein f1 (Foxf1) is significantly increased in the vertebrae of ovariectomized mice [37]. Compared with the control group, knocking down Foxf1 significantly increased ALP activity and the number of mineralized nodules, while elevating the expression levels of Runx2, ALP, Osx, OCN, and Col1a1 mRNA. Furthermore, the expression levels of β-catenin mRNA and protein in BMSCs were also significantly increased [37]. Shen et al. [37] explored the effect of Foxf1 overexpression on the osteogenic potential of human bone marrow stromal stem cells. Foxf1 overexpression significantly reduced the levels of bone formation markers, such as Runx2 and Col1a1. Increased expression of Foxf1 is associated with decreased levels of bone mineral density (BMD) and bone formation markers [37].

The collective evidence indicates that non-coding RNA and Foxf1 can inhibit the expression of the key target molecule, β-catenin, by suppressing the Wnt/β-catenin signaling pathway. This suppression inhibits downstream ALP activity and the expression of osteogenesis-related markers, such as Runx2, OPN, OCN, and Col1a1. The classical Wnt/β-catenin signaling pathway is involved in the occurrence, development, and prognosis of osteoporosis. However, its complex and widespread mechanisms require further investigation to elucidate the regulatory network and aid in the prevention and treatment of osteoporosis.

### Non-classical Wnt signal pathway

As mentioned previously, the non-classical Wnt signaling pathways mainly include the Wnt/PCP and Wnt/Ca2+ signaling pathways. Notably, the Wnt/PCP signaling pathway coordinates cell polarization, with downstream activation of c-Jun N-terminal kinase by Dsh, Rac, and small GTPases [38, 39]. However, in the Wnt/Ca2+ signaling pathway, the Wnt ligands bind to FZD and Ror2, activating Ca2+-sensitive signaling molecules through calmodulin-dependent protein kinase II (CaMKII) or calcineurin phosphatase [40]. These activations increase the intracellular Ca2+ concentration, regulating cell motility and adhesion [40] (Figure 1).

Lin et al. [41] described significant increases in the mRNA levels of osteogenic markers ALP, Runx2, and OCN upon treatment of BMSCs with JTE013, an antagonist of sphingosine-1-phosphate receptor 2. In addition, compared to the control, treatment with 2–8 µM JTE013 significantly increased the expression of phospho-phospholipase Cγ1 (p-PLCγ1) and phospho-protein kinase C (p-PKC) in BMSCs, while treatment with 1–4 µM significantly increased the expression of p-CaMKII [41]. Moreover, treatment with 1–8 µM JTE013 significantly increased calcium release in BMSCs [41]. These findings indicate the important role of the Wnt/Ca2+ signaling pathway in osteogenesis and suggest that JTE013 promotes osteogenesis through this pathway.

Li et al. [42] reported that miR-154-5p significantly inhibits ALP activity in adipose-derived MSCs (ADSCs), while miR-154-5p antisense oligonucleotide (ASO-154-5p) significantly increases ALP activity. These results were confirmed by Alizarin Red staining [42]. Data from qRT-PCR and western blot analyses demonstrated that overexpression of miR-154-5p significantly downregulates the expression of OCN, type I collagen (Col1), OPN, ALP, and Runx2, whereas its inhibition upregulates the expression of these osteogenic markers in ADSCs [42]. These results indicate that miR-154-5p expression inhibits osteogenic differentiation in ADSCs, while ASO-154-5p promotes osteogenic differentiation by inhibiting miR-154-5p [42].

In this study, the expression of Wnt11 decreased in ADSCs treated with miR-154-5p, and the overexpression of miR-154-5p significantly inhibited the activation of Ras homolog gene family member A (RhoA) and Rho-associated coiled helix kinase II (ROCKII). However, treatment with ASO-154-5p reversed these results [42]. These findings indicate that Wnt11 is a regulator of the non-classical Wnt/PCP signaling pathway and that ASO-154-5p promotes osteogenic differentiation by negatively regulating Wnt11 via miR-154-5p inhibition.

A previous study [43] found that overexpression of miR-26a-5p decreases the expression of osteogenic markers OCN, Col1, Runx2, ALP, and Osx in ADSCs, while an miR-26a-5p antagonist reverses these effects. ALP and Alizarin Red S (ARS) staining revealed that differentiation of ADSCs in the miR-26a-5p group significantly decreased, whereas differentiation in the miR-26a-5p antagonist group significantly increased, suggesting that overexpression of miR-26a-5p inhibits osteogenic differentiation of ADSCs [43].

These results confirmed that miR-26a-5p overexpression downregulates the protein expression of Wnt5a in ADSCs and significantly reduces the expression of CaMKII and osteogenesis-related marker protein Col1, which are key factors in the Wnt/Ca2+ signaling pathway [43]. The collective evidence demonstrates that miR-26a-5p inhibits osteogenic differentiation of ADSCs by directly downregulating Wnt5a expression [43]. The expression level of PKC, another key factor in the Wnt/Ca2+ signaling pathway, decreased by 58% in the miR-26a-5p group and significantly increased in the miR-26a-5p antagonist group [43]. Additionally, the level of intracellular Ca2+ significantly decreased in the miR-26a-5p group and significantly increased in the anti-miR-26a-5p antagonist group [43]. These results further suggest that miR-26a-5p inhibits osteogenic differentiation of ADSCs by regulating PKC expression, another key factor related to calcium in the Wnt/Ca2+ signaling pathway.

The available evidence highlights that the osteogenic differentiation mechanism of BMSCs via the Wnt signaling pathway is a complex regulatory system. A better understanding of this pathway may aid in the development of new tools for more effective diagnosis and treatment of osteoporosis. Furthermore, the collective data indicate that overexpression of certain miRNAs can inhibit the expression of downstream osteogenesis-related markers OCN, Col1, Runx2, ALP, and Osx by inhibiting key proteins, such as RhoA, ROCKII, CaMKII, and PKC in the non-classical Wnt/Ca2+ signaling pathway, thereby promoting the progression of osteoporosis. A better understanding of the regulatory mechanism of the non-classical Wnt pathway in osteoporosis progression at the molecular level will enable more effective diagnosis and treatment of this disease.

### Inhibitors of Wnt signaling pathway

The primary inhibitors of the Wnt signaling pathway include sclerostin, DKK1, WIF1, and SFRP [44]. Sclerostin and DKK1 can competitively bind to LRP5/6, a co-receptor in the Wnt signaling pathway, and inactivate it by regulating the transcription of downstream target genes, eventually inducing osteoporosis. SFRP and WIF1 can directly interact with Wnt ligands, blocking the binding of the ligands to the receptor and inactivating the Wnt signaling pathway by regulating the transcription of downstream target genes, ultimately leading to the occurrence and progression of osteoporosis [45–47].

## Sclerostin

**Figure 2.**
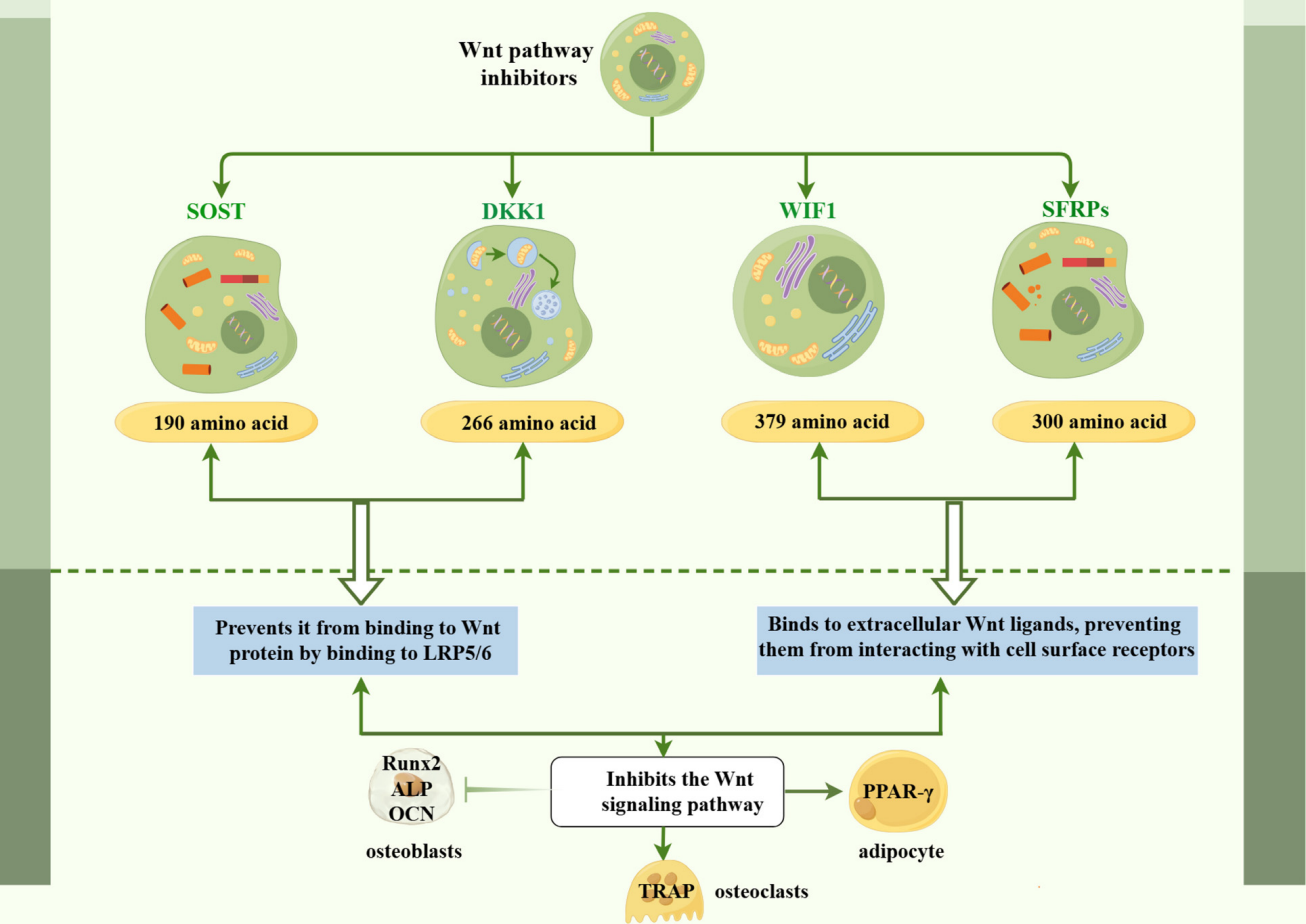
**Inhibition of Wnt/β-catenin signaling pathway by Wnt signaling pathway inhibitors.** Inhibitors of the Wnt signaling pathway mainly include sclerostin, DKK1, WIF1, and SFRPs. Among them, sclerostin and DKK1 can competitively bind to the co-receptor LRP5/6 in the Wnt signaling pathway and inactivate it by regulating the transcription of downstream target genes. SFRPs and WIF1 can directly interact with Wnt ligands, thereby blocking their binding and subsequently inactivating the Wnt signaling pathway through regulation of the transcription of downstream target genes, ultimately leading to the occurrence and development of osteoporosis. SFRP: Secreted frizzled-related proteins; DKK1: Dickkopf-1; LRP5/6: Lipoprotein receptor-related protein 5/6; WIF1: WNT inhibitory factor 1.

The sclerostin protein encodes a secreted glycoprotein composed of 190 amino acids that is primarily produced by osteocytes [48–51]. Sclerostin is a key molecule in the Wnt signaling pathway and plays a pivotal role in the regulation of osteoblast and osteoclast activity. It mainly participates in the negative regulation of bone formation [46, 48–51]. Sclerostin acts as an antagonist of the Wnt signaling pathway [52] and prevents binding to the Wnt ligand by competitively binding to LRP5/6 [53]. This antagonistic action inhibits Wnt signaling activation [54], which suppresses the differentiation of osteoblasts (Figure 2). In vitro experiments revealed that sclerostin significantly reduced the ability of human MSCs (hMSCs) to differentiate into osteoblasts in a dose-dependent manner, markedly reduced ALP activity, and increased caspase activity in hMSC osteoblasts [55]. Staining of apoptotic nuclei revealed a significant increase in the number of apoptotic cells following sclerostin treatment. These findings indicate that sclerostin selectively enhances the apoptosis of hMSC osteoblasts, possibly by inhibiting osteogenic differentiation [55].

Another study [56] revealed that the RNA levels of the osteoblast markers COL1A1 and ALP significantly decreased in hMSCs treated with sclerostin, and that sclerostin significantly reduced the proliferation and mineral deposition of hMSCs in a dose-dependent manner [56]. The authors further reported that, compared with wild-type mice, sclerostin overexpression disrupted the bone structure of sclerostin transgenic mice, induced thinning of the bone cortex, decreased the number of trabeculae, and weakened fracture resistance in the vertebrae and femur of sclerostin transgenic mice [56]. Histomorphometric analysis demonstrated that sclerostin overexpression significantly reduced the bone formation rate in sclerostin transgenic mice compared to that in wild-type mice [56]. These results indicate that sclerostin significantly reduced hMSC osteoblast markers, hMSC proliferation, and mineral deposition, and its overexpression reduced the bone formation rate and aggravated bone microstructure damage in sclerostin transgenic mice [56].

Li et al. [57] found that the BMD, trabecular bone, bone volume, and bone volume fraction in various bone regions (skull, axial bone, ribs, pelvis, and long bone) as well as serum OCN levels were significantly increased in sclerostin knockout mice compared to those in wild-type mice [57]. In addition, micro-computed tomography revealed that the thickness, area, and periosteal circumference of sclerostin knockout mice significantly increased. Based on the findings of this study [57], the mineralization area, expressed as the ratio of the mineralizing surface and bone surface (MS/BS), mineral attachment rate (MAR), and bone formation rate, were significantly increased in sclerostin knockout mice compared to those in wild-type mice [57]. Gao et al. [58] isolated rat BMSCs and overexpressed or knocked down sclerostin via transfection. The overexpression of sclerostin inhibited the proliferation of rat BMSCs, the activity of ALP, the expression of key molecules (β-catenin and p-GSK-3β) in the Wnt/β-catenin signaling pathway, and the expression of osteogenic genes Runx2 and c-myc. Knockdown of sclerostin resulted in opposite findings [58]. These results suggest that the upregulation of sclerostin can inhibit the Wnt/β-catenin signaling pathway in BMSCs, thereby inhibiting the expression of osteogenic markers.

In summary, sclerostin downregulates the expression of osteoblast markers, including COL1A1 and ALP, and upregulates the expression of the caspase pro-apoptotic protein in the Wnt signaling pathway by inhibiting Wnt-LRP5/6 receptor binding. This inhibition suppresses osteogenesis, promotes osteoblast apoptosis, and exacerbates the destruction of bone microstructure in osteoporosis. Thus, sclerostin may be an effective therapeutic target for osteoporosis.

### DKK1

The DKK family comprises four glycoproteins (DKK1-4), each containing 255–350 amino acids [59]. DKK1 has the most extensive function and is mainly expressed in osteocytes, osteoblasts, skin, placenta, and prostate endothelial cells. It consists of five domains: signal sequence, linker 1, amino-terminal cysteine-rich domain, linker 2, and carboxyl-terminal cysteine-rich domain. DKK1 inhibits the Wnt/β-catenin signaling pathway and prevents binding to the Wnt protein by competitively binding to LRP5/6, thereby blocking the conduction of the Wnt signaling pathway [53, 60] (Figure 2).

Li et al. [61] found that overexpression of DKK1 in mice resulted in a 20% reduction in the BMD of the proximal tibial metaphysis, a significant decrease in the trabeculae of the vertebrae and long bones, and marked thinning of the bone cortex compared to control mice [61]. Histomorphometric analysis revealed that the bone volume/tissue volume (BV/TV) decreased by 44%, and the percentage of osteoblast surface to bone surface significantly decreased by 49% in mice overexpressing DKK1 [61]. Bone mineralization surface (MS), bone formation rate (BFR)/TV, trabecular bone volume fraction (BVF), trabecular number, and trabecular thickness were also significantly decreased in these mice [61]. These results indicate that DKK1 overexpression aggravates the degradation of bone microstructure in mice by reducing the BMD of the proximal tibial metaphysis and the number of trabeculae in the vertebrae and long bones [61].

Another study [62] found that when DKK1 was transfected into osteoblasts, the expression levels of the Axin2 and ALP bone formation markers were significantly inhibited; this result was confirmed by Oil Red O staining. Alizarin Red fluorescence results also revealed that recombinant DKK1 (rmDkk1) significantly reduced the fluorescence intensity of matrix mineralization and ALP levels in mouse embryonic osteoblasts (MC3T3-E1) [62]. Compared with control mice, the number of osteoblasts and bone formation markers, such as ALP, Runx2, OC, and Osx, significantly increased in DKK1-deficient mice [62]. The MS and MAR of DKK1-deficient mice also increased by twofold, and the trabecular BFR increased by fourfold [62]. These results confirm that the downregulation of DKK1 can activate the expression of osteoblast markers in the Wnt signaling pathway to promote bone formation and inhibit bone resorption in osteoporosis. DKK1 may be another effective target for the treatment of osteoporosis.

### WIF1

WIF1 is a protein composed of 379 amino acid residues, with an N-terminal signal sequence of 28 amino acid residues, a WIF domain of approximately 150 amino acids, five epidermal growth factor (EGF)-like repeat sequences, and a C-terminal hydrophilic domain of 45 amino acids [63]. As an inhibitor of the Wnt signaling pathway [64], WIF1 can directly bind to Wnt, blocking its interaction with the cell surface receptor FZD and ultimately inhibiting the transmission of this signaling pathway [65–68] (Figure 2). According to Bennett et al. [69], WIF1 is a negative regulator of osteoblast differentiation, and its overexpression can stimulate adipogenesis by inhibiting the Wnt signaling pathway, thereby inhibiting bone formation. Cho et al. [70] and other researchers found that the overexpression of WIF1 can significantly inhibit ALP activity and ALP staining intensity in C3H10T1/2 cells. In addition, WIF1 overexpression significantly inhibited Runx2 expression and mRNA levels of type I collagen, ALP, and osteocalcin in C3H10T1/2 cells [70].

The role of WIF1 in the adipogenic differentiation of C3H10T1/2 cells was also evaluated. When C3H10T1/2 cells were induced with lipogenic medium, the mRNA and protein expression of WIF1 significantly increased. Oil Red O staining revealed that treatment with WIF1 significantly enhanced adipogenic production in C3H10T1/2 cells [70]. Thus, WIF1 overexpression can significantly inhibit the activity of ALP, staining intensity of ALP, and expression of bone formation markers in C3H10T1/2 cells, while promoting adipogenic differentiation in these cells [70]. Wei et al. [71] also found that the mRNA and protein expression of OPN, OCN, and Runx2 significantly decreased in the bone marrow stromal cells of patients with osteoporosis compared to those in control individuals. ALP in the BMSCs of patients with osteoporosis decreased significantly compared with control individuals [71]. When WIF1 was overexpressed, the expression levels of Wnt and β-catenin decreased significantly compared with those in the control group [71].

According to another study [72], the protein expression of β-catenin in chondrocytes treated with Wnt3a increased significantly; however, WIF1 could effectively block this accumulation. Wnt3a also significantly increased the transcriptional activity of TCF/LEF in chondrocytes, which was significantly inhibited by WIF1 [72]. Therefore, WIF1 can inhibit the expression of β-catenin, a key molecule in the Wnt signaling pathway, and the transcriptional activity of TCF/LEF by binding to the Wnt ligand, thereby inhibiting Runx2. This results in the suppression of osteogenic markers, such as type I collagen, ALP, and osteocalcin while promoting adipogenic differentiation. WIF1 could therefore serve as a key target in the research and development of therapies for osteoporosis.

### SFRPs

SFRPs are soluble proteins composed of approximately 300 amino acids, including a highly homologous N-terminal cysteine-rich domain (CRD) and a smaller hydrophilic C-terminal domain (NRT) [73–76]. Both CRD and NRT domains can bind to Wnt signaling molecules, with different SFRP proteins binding to different subgroups of Wnt molecules [76]. In addition, SFRPs consist of five protein families (SFRP1–SFRP5), which are divided into two subfamilies based on sequence homology. One subfamily consists of SFRP1, 2, and 5, while the other subfamily consists of SFRP3 and 4 [76, 77]. SFRPs regulate bone metabolism [78]. Some studies [53, 79] have reported that SFRPs, as antagonists of the Wnt signaling pathway, can bind to Wnt and inhibit its interaction with receptors, thereby inactivating the Wnt signaling pathway by regulating the transcription of downstream target genes, leading to the occurrence and development of osteoporosis (Figure 2).

Bodine et al. [80] found that the body fat rate of SFRP1-deficient mice decreased by 22%, the MAR of the distal femur trabecular bone increased by 32%, connection density increased by 29%–47%, the number of trabecular bones increased by 18%–25%, and the thickness of the trabecular bone increased by 4%–19%, compared with the values of control mice [80]. The lack of SFRP1 resulted in a 4%–56% decrease in the number of apoptotic osteoblasts and osteocytes, whereas a decrease in apoptotic cells led to an 18% increase in skull thickness and a 5% increase in the number of osteocytes [80]. Another study found that the overexpression of SFRP2 significantly decreased the expression of the Axin and OCN osteogenic markers in BMSCs compared to that of the control cells (81). qRT-PCR further confirmed that SFRP2 significantly decreased the expression of OCN and Runx2 [81]. These findings indicate that SFRP2 overexpression could inhibit osteogenic differentiation.

In a model of dexamethasone-induced osteoporosis, He and Gu [82] confirmed the upregulation of SFRP5. The overexpression of SFRP5 downregulated the protein expression of Wnt and β-catenin within the Wnt signaling pathway and further downregulated the expression of the Runx2, ALP, and OPN osteogenesis-related markers [82]. These results were confirmed via Alizarin Red S staining [82]. These findings implicate SFRP1, SFRP2, and SFRP5 as key targets of the Wnt signaling pathway to regulate bone metabolism in osteoporosis. However, SFRP3 and SFRP4 exhibited opposite effects on the osteoblast differentiation of hMSCs [83]. SFRP3 promotes the osteoblast differentiation of hMSCs, whereas SFRP4 inhibits it [83]. The authors cultured hMSCs in osteoinductive medium; the mRNA and protein expression of SFRP3 gradually increased, while the expression of SFRP4 was significantly inhibited on days 4, 7, and 14 of culture [83].

They further reported that the treatment of hMSCs with recombinant SFRP3 did not affect the activity of ALP and the formation of mineralized nodules, while treatment with recombinant SFRP4 significantly reduced the activity of ALP and inhibited the formation of mineralized nodules [83]. Finally, the ALP activity of hMSCs was inhibited when SFRP3 activity was specifically abrogated by treatment with small interfering (si)SFRP3, but it increased following treatment with SFRP4 [83]. The collective evidence indicates that the deletion of SFRP1, 2, 4, and 5 can significantly improve the bone microstructure damage induced by osteoporosis, which may be achieved by markedly upregulating the expression of the Wnt and β-catenin proteins and downstream osteogenesis-related markers in the Wnt/β-catenin signaling pathway, thereby promoting osteogenesis in osteoporosis. The overexpression of SFRP1, 2, 4, and 5 can inhibit osteogenesis, whereas SFRP3 overexpression can promote the osteogenic differentiation of hMSCs. Therefore, whether the specific regulatory mechanism of SFRPs in the occurrence and development of osteoporosis aims to promote bone differentiation or inhibit osteogenic differentiation is a complex question. Overall, more in-depth studies are needed.

## Regulation of osteoporosis via the knockout of wnt signaling pathway inhibitors

### Regulatory effect of sclerostin knockout on osteoporosis

Sclerostin is an inhibitor of the Wnt signaling pathway. In a mouse model of bone defects, McGee-Lawrence [84] confirmed that the bone mass of sclerostin-/- mice significantly increased, the BV fraction of the defect site increased by 49%, the defect diameter significantly decreased, and trabecular thickness (Tb.Th) significantly increased. Furthermore, the expression of the Runx2 osteogenesis-related gene significantly increased in a mouse model of bone defects relative to the values in sclerostin+/+ mice [84]. These results indicate that blocking sclerostin could promote the expression of osteogenic genes, thereby significantly improving the bone microstructure.

Zhang et al. [85] confirmed significant increases in the skull BMD, BV, and BV/TV of a mouse osteolytic model upon blocking of sclerostin. This blocking significantly increased the activity of ALP, the expression of β-catenin on the skull bone surface, the number of mineralized nodules, and the Ca^2+^ level in a mouse osteolytic model [85]. Therefore, blocking sclerostin may play a role in promoting bone formation and alleviating bone microstructure damage by activating the Wnt/β-catenin signaling cascade. Jiao et al. [86] found that the inhibition of sclerostin significantly increased BMD and BV/TV in the skull, increased the expression of key Wnt/β-catenin signaling pathway proteins, such as β-catenin and OPG, and decreased the expression of osteoclast-related markers, including nuclear factor of activated T cells 1, cathepsin K, and TRAP [86]. Furthermore, the number of TRAP-positive cells was significantly diminished in the mouse osteolytic model [86]. These results indicate that blocking sclerostin can promote osteogenesis and inhibit osteoclast differentiation and bone resorption by affecting the expression of key proteins in the Wnt/β-catenin signaling pathway, thus regulating osteoclast-osteoblast marker molecules.

**Figure 3.**
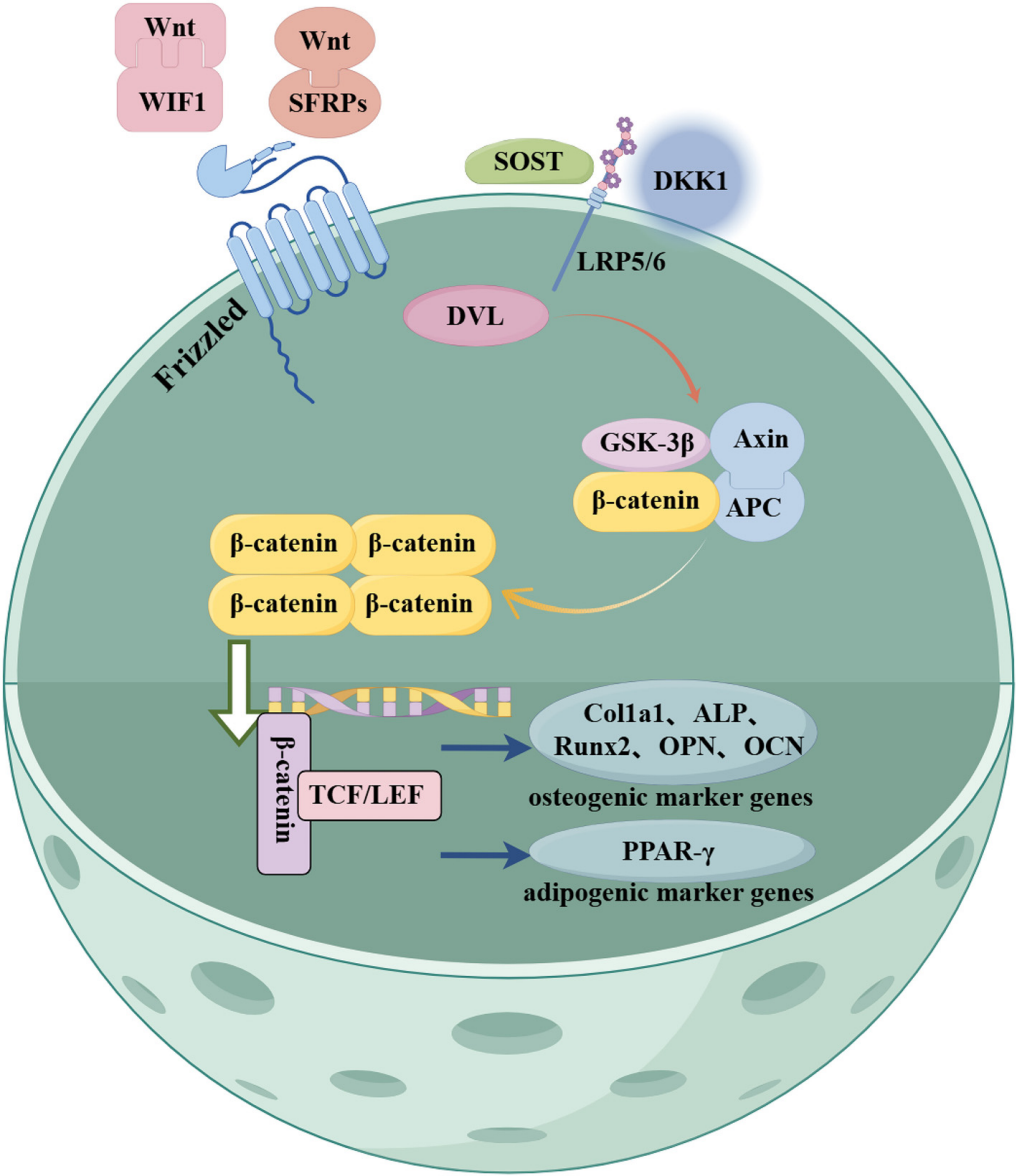
**Wnt signaling pathway inhibitors.** Inhibitors of the Wnt signaling pathway mainly include sclerostin, DKK1, WIF1, and SFRPs. These proteins are composed of varying amounts of amino acids and primarily inhibit the conduction of the Wnt signaling pathway by binding to the ligand cell membrane Frizzled (FZD) receptor and low-density LRP5/6 of the Wnt signaling pathway. DKK1: Dickkopf-1; LRP5/6: Lipoprotein receptor-related protein 5/6; WIF1: WNT inhibitory factor 1.

Therefore, sclerostin is a key target in the regulation of the Wnt/β-catenin pathway. Blocking sclerostin can promote the expression of osteoblast marker molecules by activating the Wnt/β-catenin pathway and can inhibit the expression of osteoclast differentiation and bone resorption markers, thereby maintaining the dynamic balance between osteoblasts and osteoclasts, and ultimately counteracting osteoporosis (Figure 3). In summary, SOST may become an effective target for future osteoporosis drug research and development and may be a key molecule in revealing the pathogenesis of osteoporosis.

### Regulation of osteoporosis via the knockout of DKK1

Heiland et al. [87] found that, compared to wild-type mice, tumor necrosis factor transgenic mice (hTNFtg) showed a significant increase in OPG mRNA expression and a significant decrease in OCN mRNA expression. However, after knockout of DKK1, OPG mRNA expression in hTNFtg mice decreased significantly, and the mRNA expression of OCN increased significantly, indicating that osteoclast activity was inhibited and osteoblast-mediated bone formation was enhanced [87]. In addition, immunohistochemistry revealed that, compared with wild-type mice, there was almost no expression of β-catenin in the trabecular bone of hTNFtg mice. However, after the knockout of DKK1, the expression of β-catenin in hTNFtg mice significantly increased. These findings indicate that the knockout of DKK1 can enhance bone formation and inhibit bone resorption in hTNFtg mice [87].

The same study also found [87] that, compared with wild-type mice, the number of bone trabeculae, trabecular thickness, matrix deposition rate (MAR), and bone surface covered by osteoblasts in hTNFtg mice were significantly reduced. However, after DKK1 knockout, the number of bone trabeculae, trabecular thickness, MAR, and bone surface covered by osteoblasts in hTNFtg mice showed a significant increasing trend, which correlated with the dose of the anti-DKK1 antibody [87]. In addition, Colditz et al. [88] found that, compared with Cre female and male mice, the BV/TV, Tb.N, Tb.Th, Ct.Th, and other indicators in Cre+ female and male mice were significantly increased, while Tb.Sp, serum DKK1 content, and femoral DKK1 mRNA expression were significantly decreased. There was no significant difference in serum CTX content [88]. Both female and male Cre+ mice showed significant increases in Procollagen type 1 N-propeptide (PINP) and OPG content, an increase in the bone formation rate/bone surface (BF/BS) ratio, a significant decrease in the number of osteoclasts/bone perimeter (N.Oc/B.Pm) ratio, and a significant decrease in RANKL content [88]. The study also reported that, compared with Cre female mice, the MS/BS ratio in Cre+ female mice was significantly increased, and the MAR was significantly elevated. Compared with the Cre female mice of Dkk1-Osx and Dkk1-Dmp1, Cre+ female mice of Dkk1-Osx and Dkk1-Dmp1 showed similar trends in BV/TV, Tb.N, Tb.Th, Ct.Th, and Tb.Sp. Serum DKK1 content, femoral tissue DKK1 mRNA expression, serum carboxy-terminal collagen crosslinks content, BF/BS ratio, N.Oc/B.Pm ratio, RANKL content, and MS/BS ratio exhibited similar changes to those in Cre+ female mice [88].

These results confirm that the deficiency of DKK1 affects bone resorption by reducing the number of osteoclasts but does not affect the serum level of the carboxy-terminal collagen crosslinks bone resorption marker. Other studies [89] have also found that the expressions of the Runx2 and OCN osteogenic markers in rats with glucocorticoid-induced osteoporosis were significantly downregulated, while the expression levels of key proteins GSK3β and β-catenin in the Wnt/β-catenin signaling pathway were significantly reduced [89]. The authors also described that the knockdown of DKK1 significantly upregulated the expression of the Runx2 and OCN osteogenic markers, while the expression levels of key proteins GSK3β and β-catenin were significantly increased [89] (Figure 3).

These findings indicate that the knockout of DKK1 can prevent bone loss. Therefore, inhibiting the expression of DKK1 in bone tissue may be an effective method for treating osteoporosis.

### Regulatory effect of WIF1 knockout on osteoporosis

The mRNA and protein levels of WIF1 were reportedly significantly reduced compared with those of the control when osteogenic medium (OM) containing ascorbic acid and β-glycerophosphate was used to induce osteoblast differentiation of mesenchymal C3H10T1/2 cells [77]. However, when lipogenic medium was used to induce lipogenesis, the mRNA and protein expressions of WIF1 significantly increased compared to those of the control, contrary to the results obtained during osteoblast formation [77]. Furthermore, when WIF1 production was blocked using siWIF1, RT-PCR revealed that the mRNA level of WIF1 was reduced by 37% and 70% using 20 and 50-nM siRNA duplexes, respectively, to target WIF1. The ALP activity of mesenchymal C3H10T1/2 cells also increased in a dose-dependent manner. These results indicate that the blocking of WIF1 plays a positive role in regulating osteoblast differentiation of mesenchymal C3H10T1/2 cells [77].

Liang et al. [90] found that gossypol significantly inhibited the level of WIF1 in a mouse model of osteoporosis, significantly upregulated the expression levels of key proteins (Wnt, β-catenin, CK1, and Axin) in the Wnt signaling pathway, and upregulated the levels of osteogenesis-related markers (OCN and OPG) in the mice [90]. These results confirm that inhibiting WIF1 can activate the Wnt-β-catenin signaling pathway to promote osteoblast differentiation in mice with osteoporosis (Figure 3). Collectively, these findings indicate that the expression of WIF1 is a key target for transcriptional activity in the Wnt-β-catenin signaling pathway. Ongoing efforts are aimed at developing WIF1 as a target for drug screening. It is anticipated that more effective anti-osteoporotic drugs will become a popular research topic.

### Regulatory effect of SFRP knockout on osteoporosis

Wang et al. [91] found that knockdown of the expression of SFRP1 significantly increased the expression levels of β-catenin protein and Runx2 in the femoral tissue of rats with glucocorticoid-induced osteoporosis, while upregulating the expression of SFRP1 resulted in the opposite effect. Knockdown of SFRP1 significantly inhibited chondrocyte apoptosis, increased mineral density, biomechanical properties, and trabecular and cortical bone mass. The overexpression of SFRP1 reversed these effects [91]. These results indicate that knockdown of the expression of SFRP1 can promote bone formation and prevent bone loss by activating the Wnt/β-catenin signaling pathway in rats with glucocorticoid-induced osteoporosis. Thus, SFRP1 could be the basis of an effective strategy for preventing osteoporosis.

Another study demonstrated that the SFRP1 inhibitor diarylsulfonylsulfonamide can stimulate the Wnt/β-catenin signaling pathway by inhibiting the expression of SFRP1 in U2-OS human osteosarcoma cells [92]. Knockout of SFRP1 reduced apoptosis of U2-OS human osteosarcoma cells by approximately 50% and increased the mineral attachment rate of bone trabeculae by approximately 30%. These findings indicate that knockout of SFRP1 can inhibit osteoblast apoptosis in U2-OS human osteosarcoma cells [92]. Oshima et al. [93] found that SFRP2 knockout significantly enhanced ALP activity and promoted the formation of mineralized nodules in mice with multiple myeloma. These findings corroborated the promoting effect of SFRP2 blockade on osteogenic differentiation [93]. In another study, the XAV939 inhibitor of the Wnt/β-catenin signaling pathway inhibited the activity of ALP in hMSCs cultured in osteoinductive medium [83]. PCR microarray analysis revealed that the expression levels of DIXDC1, FZD5, WISP1, and SFRP3 genes in the Wnt/β-catenin signaling pathway were significantly upregulated, while those of CCND2 and SFRP4 were significantly downregulated [83]. These results suggest that SFRP3 and SFRP4 are involved in the regulation of the Wnt/β-catenin signaling pathway [83].

These results further indicate that the Wnt signaling pathway is involved in the osteogenic differentiation of hMSCs. SFRPs control the formation of osteoblasts by affecting the classical and non-classical Wnt pathways, and SFRPs may play a role in the prevention and treatment of osteoporosis. After the knockout of SFRP5, the protein expression levels of Wnt and β-catenin were significantly upregulated in rats with dexamethasone-induced osteoporosis [82]. RT-qPCR revealed that after the downregulation of SFRP5, the mRNA and protein levels of Runx2, ALP, and OPN in the femur and tibia of rats with dexamethasone-induced osteoporosis were significantly increased [82]. Alizarin Red S staining further confirmed that SFRP5 knockout significantly promoted the osteogenic differentiation of BMSCs in dexamethasone-induced osteoporotic rats [82].

In vitro experiments further confirmed that SFRP5 knockout significantly promoted the osteogenic differentiation of BMSCs by regulating the Wnt/β-catenin signaling pathway [82]. Overall, in most cases, blocking the expression of SFRPs can promote the expression of osteogenic differentiation markers in the Wnt/β-catenin signaling pathway in osteoporosis, thereby promoting osteogenic differentiation. However, blocking the upregulation of SFRP3 can inhibit the expression of osteogenic differentiation markers within the Wnt/β-catenin signaling pathway, ultimately inhibiting osteogenic differentiation. The regulatory mechanism of SFRP blockers in osteogenic differentiation during osteoporosis must be further verified and explored through in vivo and in vitro studies (Figure 3).

## Targeted therapy

### miR-203

miRNA is a non-coding, small RNA containing approximately 19–24 nucleotides [94]. Various miRNAs play crucial regulatory roles in the differentiation and development of cells and tissues [95]. In recent years, miRNA research on osteoporosis has become a focal point. Qiao et al. [96] constructed a rat model of osteoporosis and transfected miR-203 into MSCs to overexpress miR-203. The luciferase gene assay results showed that the expression level of DKK1 (luciferase activity) in rats co-transfected with the miR-203 mimic and wild-type DKK1 was significantly lower than that in the normal control group [96].

Compared with the control group, the BMD and BV/TV of the miR-203 inhibitor group and OVX group rats were significantly decreased, while the femoral bone resorption parameters (Ob.S/BS) and the number of osteoclasts per bone surface (N.Oc/B.Pm) in the ovariectomized rats injected with the miR-203 inhibitor were higher than those in the normal control group [96]. In addition, clinical trials revealed that the overexpression of miR-203 enhanced the expression levels of the ALP, Bglap, and Runx2 bone-related genes in postmenopausal patients with osteoporosis and reduced the expression levels of peroxisome proliferator-activated receptor-gamma (PPARγ) and lipoprotein lipase (LPL) genes associated with fat production [96]. These results indicate that DKK1 is the target gene of miR-203. miR-203 can promote the expression of osteogenic marker molecules and inhibit the expression of adipogenic marker molecules by targeting and downregulating DKK1, thereby regulating the dynamic balance between osteogenesis and adipogenesis in osteoporosis. Additionally, in another study, serum miR-203 levels in patients with osteoporosis were found to be lower than normal levels [97]. The authors also examined the effect of miR-203 on ALP activity in MSCs from patients with osteoporosis. The results showed that decreased miR-203 expression significantly reduced ALP activity in these patients, while the overexpression of miR-203 significantly enhanced ALP activity [97]. In the same study, DKK1 was selected as the target gene of miR-203 for experimentation [97]. The overexpression of miR-203 significantly downregulated the expression of DKK1 in patients with osteoporosis, while the downregulation of miR-203 produced the opposite trend. The effect of miR-203 on the osteogenic differentiation of MSCs in patients with osteoporosis, by inhibiting DKK1, was also studied [97]. The results showed that the overexpression of miR-203 significantly upregulated the expression of osteoblast-related genes ALP and Runx2, as well as the expression of osteoblast-related proteins Runx2, OCN, and OPN in patients. Conversely, overexpression of DKK1 reversed the expression of these markers [97].

**Figure 4.**
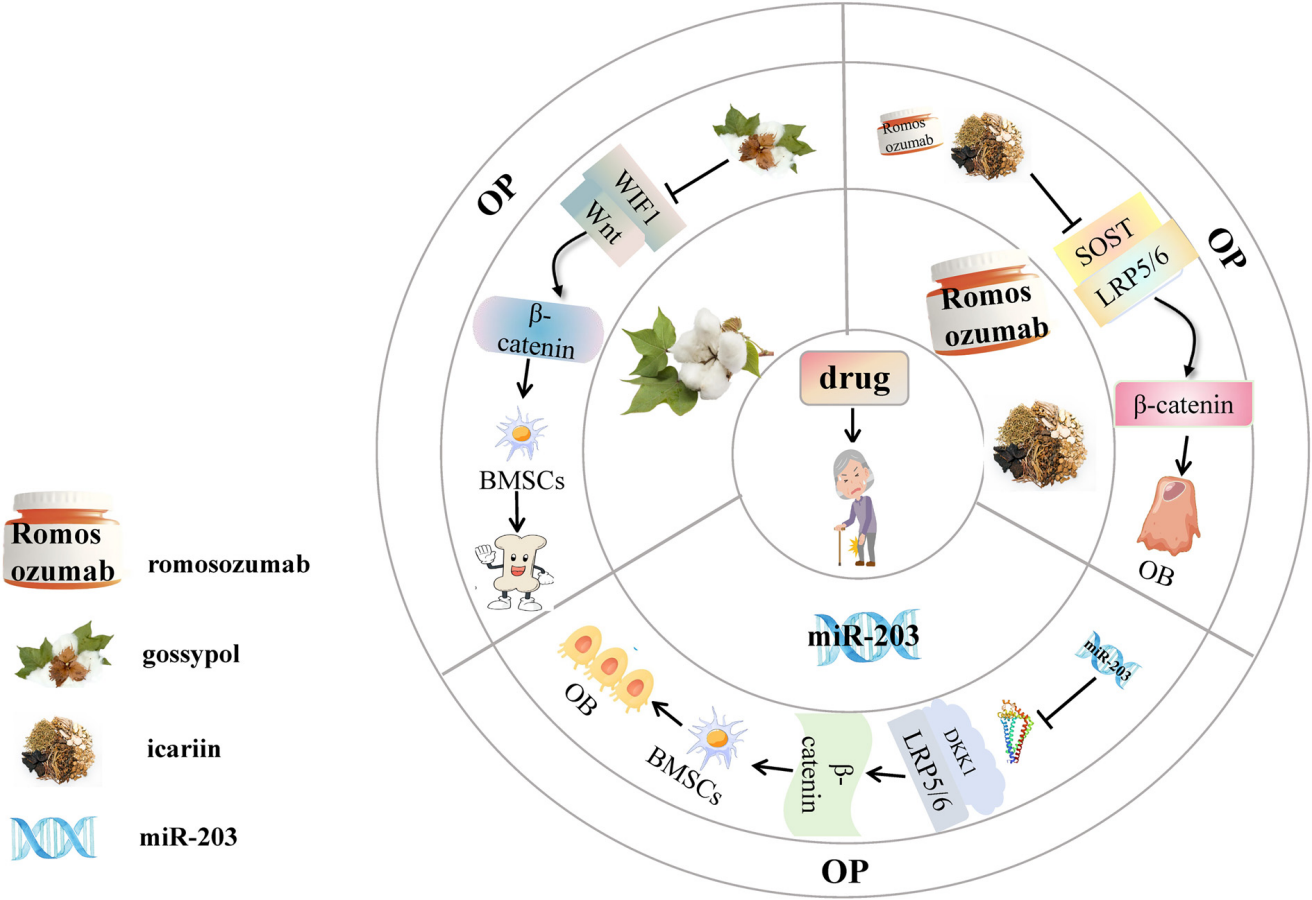
**Targeted therapy for osteoporosis.** miR-203 activates the Wnt/β-catenin signaling pathway by inhibiting the expression of DKK1. Romosozumab promotes the conduction of the Wnt/β-catenin signaling pathway by preventing sclerostin from binding to LRP5/6. Icariin and gossypol can inhibit the interaction between WIF1 and Wnt protein, thereby regulating the transcription of downstream target genes. This activation of the Wnt/β-catenin signaling pathway ultimately alleviates the occurrence and development of osteoporosis. DKK1: Dickkopf-1; LRP5/6: Lipoprotein receptor-related protein 5/6; WIF1: WNT inhibitory factor 1.

These findings indicate that miR-203 can upregulate the expression of osteogenic markers by inhibiting DKK1, thereby promoting the osteogenic differentiation of BMSCs. Therefore, targeting miR-203 and studying the mechanisms of DKK1 regulation could provide valuable insights into the mechanisms of osteoporosis (Figure 4).

### Romosozumab

Romosozumab is a humanized monoclonal antibody targeting sclerostin. The antibody binds to sclerostin, inhibits its activity, and promotes the binding of Wnt ligand to its co-receptor, thereby increasing bone formation and BMD [98]. Subcutaneous or intravenous administration significantly increases serum levels of bone formation markers, such as P1NP, ALP, and osteocalcin, and significantly decreases the level of serum C-terminal peptide (CTX), a bone resorption marker, in patients with osteoporosis [98]. By day 85 of administration, romosozumab increased lumbar BMD by 5.3% and total hip BMD by 2.8% in patients with osteoporosis compared to healthy controls [98]. Additionally, when the dosage of romosozumab was increased to 210 mg/kg, serum P1NP levels increased by 66%–147%, serum CTX decreased by 15%–50%, and lumbar BMD increased by 4%–7% [98]. Therefore, the key mechanism of romosozumab in treating osteoporosis is closely related to its promotion of bone formation and inhibition of bone resorption [98].

In a phase II clinical trial, McClung et al. [99] found that the BMD of the lumbar vertebrae, total hip, and femoral neck in patients with osteoporosis significantly increased after 12 months of romosozumab administration compared to healthy controls. When the romosozumab dose was increased to 210 mg/kg, lumbar vertebrae BMD increased by 11.3%, hip joint BMD increased by 4.1%, and femoral neck BMD increased by 3.7% [99]. After six months of romosozumab treatment, the expression of P1NP, a bone turnover marker, significantly increased in patients with osteoporosis. By 12 months, P1NP and TRAP levels returned to normal [99].

In a phase III clinical trial, the risk of vertebral fractures in postmenopausal women with osteoporosis decreased by 73% after 12 months and by 75% after 24 months of romosozumab treatment at a 210 mg/kg dose [99]. These results indicate that romosozumab, a humanized monoclonal antibody targeting sclerostin, increases BMD in patients with osteoporosis by enhancing bone formation and turnover markers while inhibiting bone resorption markers. Furthermore, romosozumab markedly reduces fracture risk as the administration period increases. However, whether the regulatory effects of romosozumab on bone formation and resorption occur via the Wnt/β-catenin signaling pathway remains unclear (Figure 4).

### Icariin (ICA)

ICA is the most abundant flavonoid in Epimedium. It promotes bone formation by stimulating the proliferation and differentiation of BMSCs, playing an important role in bone regeneration and remodeling [100, 101]. Gao et al. [58] found that ICA significantly increased BMSC proliferation, osteogenic differentiation, ALP expression, and mineralized nodule formation compared to the control. Using sclerostin overexpression and short hairpin RNA (shRNA) constructs to transfect BMSCs, they observed that sclerostin overexpression significantly inhibited BMSC proliferation and ALP activity, whereas knockdown of sclerostin reversed these effects [58]. Treatment with ICA restored the osteogenic ability of BMSCs by inhibiting sclerostin overexpression in cells transfected with shRNA constructs [58].

At 4, 7, and 14 days after ICA treatment, sclerostin overexpression, as well as the expression of osteogenic genes such as Runx2, c-myc, β-catenin, and p-GSK-3β, were significantly increased [58]. These results indicate that ICA promotes bone differentiation of BMSCs by activating the Wnt/β-catenin signaling pathway. According to Wei et al. [102], ICA induces the formation of calcified nodules in hBMSCs. Alizarin Red staining revealed similar results.

In addition, compared to the control group, the ICA treatment group showed significantly upregulated activity of hBMSCs and increased expression levels of osteogenesis-related markers, including OCN, Runx2, and ALP [102]. The expression levels of OCN, Runx2, ALP, and β-catenin in hBMSCs were also significantly upregulated on days 3, 7, and 14 of ICA treatment, while sclerostin expression was significantly downregulated on days 7 and 14 [102]. Overall, ICA activates the expression of ALP and Runx2 osteogenesis-related markers via the Wnt/β-catenin signaling pathway by inhibiting sclerostin expression. This further promotes the osteogenesis of BMSCs, which may be the key mechanism underlying its therapeutic effects in osteoporosis (Figure 4).

### Gossypol

Gossypol is a natural polyphenolic compound [103] extracted from cotton seeds, roots, and stems. It exhibits various biological properties, including antiviral, antioxidant, antiparasitic, and antibacterial activities [104]. Liang et al. [105] found that administering gossypol significantly increased trabecular bone thickness, metaphyseal cortical bone thickness, and serum osteocalcin and OPG levels [105]. Gossypol also significantly enhanced medullary and cortical BMD, the expression of Wnt protein, β-catenin, and GSK-3β, as well as the mRNA levels of osteogenesis-related genes, including osteocalcin, Runx2, OPG, and COL1A1, in mice with osteoporosis compared to control mice [105]. Thus, gossypol upregulates osteogenesis-related markers in the serum of osteoporotic mice by activating the Wnt/β-catenin signaling pathway, thereby reducing bone loss.

In another study [90], gossypol administration inhibited the transcription of WIF1 in osteoporotic mice, significantly upregulated serum levels of osteocalcin, Wnt, β-catenin, OPG, and Axin, and reduced the apoptosis of MC3T3-E1 cells by 6% and TUNEL-positive cells to 56%. However, administering WIF1 inhibited the expression of Axin and myc [90]. Overexpression of WIF1 increased the apoptosis rate of MC3T3-E1 cells by 9% and doubled TUNEL-positive cells [90]. Overall, WIF1 inhibited Axin and myc expression in a dose-dependent manner, while gossypol upregulated serum osteocalcin, Wnt, β-catenin, OPG, and Axin levels by suppressing WIF1 transcription. By reducing apoptosis, gossypol promotes osteogenesis, offering a protective effect against osteoporosis. WIF1 may be the key target molecule mediating gossypol’s action (Figure 4).

## Conclusion

Osteoporosis is the most common chronic metabolic bone disease, characterized by low bone mass, decreased bone density, and deterioration of bone microstructure, which increases the risk of fragility and fractures. These factors impose a heavy economic burden on society and families. While pharmacological interventions remain the primary treatment for osteoporosis, their long-term use is associated with adverse effects. Therefore, novel therapeutic targets and drugs are needed for effective management. The Wnt signaling pathway plays a critical role in biological processes, such as cell proliferation, differentiation, apoptosis, and oxidative stress [106]. This pathway is also involved in the pathogenesis of osteoporosis [107]. Specifically, inhibitors of the Wnt signaling pathway, including sclerostin, DKK1, WIF1, and SFRP, negatively regulate bone formation during osteoporosis. These inhibitors may suppress the expression of key molecules in the Wnt/β-catenin signaling pathway, such as Wnt, β-catenin, and GSK-3β. Consequently, they inhibit osteogenic genes, including Runx2, ALP, and OCN, as well as the osteoclast-suppressing gene OPG, thereby contributing to the progression of osteoporosis. However, silencing sclerostin, DKK1, WIF1, SFRP, and other Wnt pathway inhibitors can reverse these effects, resulting in therapeutic benefits. Agents such as miR-203, romosozumab, ICA, and gossypol effectively block the expression of Wnt pathway inhibitors like sclerostin, DKK1, WIF1, and SFRP. This leads to the upregulation of key molecules in the Wnt/β-catenin signaling pathway, including Wnt, β-catenin, and GSK-3β. These agents further enhance the expression of osteogenic markers, such as Runx2, OCN, and OPN, thereby promoting bone formation. Clinical studies have demonstrated significant increases in femoral and lumbar bone density, elevated serum levels of osteogenic marker molecules (e.g., P1NP), and reduced vertebral fracture risk in female osteoporosis patients. Thus, targeting Wnt signaling pathway inhibitors, such as sclerostin, DKK1, WIF1, and SFRP, to regulate the expression of key molecules in the Wnt pathway represents a promising and safe therapeutic strategy for osteoporosis. A deeper understanding of the mechanisms underlying the regulation of the Wnt signaling pathway by these inhibitors could provide valuable insights for developing new treatments for osteoporosis.
